# Enhancing Early Detection of Contralateral Breast Cancer in Breast Cancer Survivors Using AI-Assisted Mammography

**DOI:** 10.1245/s10434-026-19348-z

**Published:** 2026-03-06

**Authors:** Ji-Jung Jung, Hong-Kyu Kim, Eunhye Kang, Eun Kyung Park, Han-Byoel Lee, Hyeong-Gon Moon, Nariya Cho, Wonshik Han

**Affiliations:** 1 Department of Surgery, Seoul National University College of Medicine, Seoul, Republic of Korea; 2 Genomic Medicine Institute, Seoul National University Medical Research Center, Seoul, Republic of Korea; 3 Cancer Research Institute, Seoul National University, Seoul, Republic of Korea; 4 Department of Radiology, Seoul National University College of Medicine, Seoul, Republic of Korea

**Keywords:** Breast cancer surveillance, Contralateral breast cancer, Artificial intelligence, Mammography, Computer-aided detection

## Abstract

**Background:**

Women with a history of breast cancer face an elevated risk of developing contralateral breast cancer (CBC). Although annual mammographic screening is recommended, its sensitivity is limited, particularly in younger women and those with dense breast tissue. This study assessed the potential of artificial intelligence (AI)-based computer-aided diagnosis (AI-CAD) to improve CBC detection in a cohort of breast cancer survivors and to enhance early CBC detection.

**Methods:**

This study included 454 women who developed CBC and 454 matched controls without recurrence. Mammograms were analyzed using AI-CAD software assigning abnormality scores from 0 to 100, with scores over 10 indicating CBC. Standalone AI predictions were compared with radiologists’ initial assessments, and its ability to detect early signs of CBC in prior mammograms was evaluated.

**Results:**

The median age of patients was 53 years; the majority (79.9%) had dense breast tissue. Standalone AI detected 271 CBC cases solely from mammography, achieving 6.2% higher sensitivity compared with radiologists (59.7% vs. 53.5%, *p* = 0.009). Notably, AI detected 66 CBC cases (14.5%) that radiologists had missed on mammographic assessment alone. With respect to earlier detection, 81 cases (29.9%) were identified on prior mammograms at a median of 13.3 (9.6–19.9) months before clinical diagnosis. Of these, 28 cases (10.3%) were detected more than 6 months before pathological confirmation, and 53 cases (19.6%) more than 1 year earlier.

**Conclusions:**

AI-CAD may enhance the detection of CBC in breast cancer survivors and facilitate earlier identification on surveillance mammography. Further studies are needed to assess its integration into surveillance mammography and clinical impact.

**Supplementary Information:**

The online version contains supplementary material available at 10.1245/s10434-026-19348-z.

Women with a history of breast cancer face a 0.3–1.0% annual risk of developing contralateral breast cancer (CBC), with a 25-year cumulative risk of 9.9%.^1,2^ This risk is approximately twice the risk of developing breast cancer in the general population.^3^ Major medical guidelines recommend annual surveillance mammogram, as early detection of asymptomatic CBC can improve prognosis compared to a diagnosis made at symptomatic onset.^4^

While mammography is the only recommended surveillance imaging method, it shows lower sensitivity and higher interval cancer rates compared with screening mammograms.^5^ Cancers can be missed due to challenges in interpreting mammograms, including breast density, history of hormone replacement therapy, and the absence of intact breast images for comparison.^6,7^ Despite these limitations, other imaging modalities, such as ultrasound and magnetic resonance imaging (MRI), are not broadly recommended owing to lack of demonstrated survival benefit.

Artificial intelligence–based computer-aided diagnosis (AI-CAD) systems have recently shown diagnostic performance comparable to that of radiologists in screening mammography.^8–11^ AI-CAD has demonstrated particular value in aiding radiologists to improve detection rate, especially in dense breast and women 50 years or younger.^12,13^ In the large-scale Mammography Screening with Artificial Intelligence trial, AI-supported reading increased cancer detection by 20% and reduced radiologists’ workload by 44% compared with standard double reading.^14^ Furthermore, AI algorithms have shown potential for individualized breast cancer risk prediction beyond traditional clinical models.^15^ However, the utility of AI-CAD in surveillance settings for breast cancer survivors remains largely unexplored.

In this study, we aimed to evaluate the performance of a commercially available AI-CAD software in detecting CBC on surveillance mammograms of breast cancer survivors. Specifically, we assessed the diagnostic accuracy of the AI-CAD software and investigated how early it could identify abnormal findings preceding CBC diagnosis. We hypothesized that the standalone AI-CAD system would demonstrate sensitivity comparable to that of baseline radiologist assessments on surveillance mammograms and would detect a subset of contralateral cancers on prior mammograms, providing measurable lead time before clinical diagnosis.

## Methods

This retrospective study was approved by the Institutional Review Board of Seoul National University Hospital (No. 2212-163-1391), and the requirement for informed consent was waived. The study was conducted in accordance with the Declaration of Helsinki and good clinical practice guidelines. Surveillance mammograms acquired between 2001 and 2024 were included for analysis. All analyses were prespecified before data extraction.

### Study Population

This retrospective study was based on the breast cancer database of Seoul National University Hospital, which includes a prospectively maintained registry of patients treated for breast cancer between 2000 and 2018. Women with a history of unilateral breast cancer who underwent at least one year of surveillance mammography after completion of primary treatment were eligible for inclusion. Routine follow-up at our institution included annual or biannual mammography with adjunctive ultrasound and, when clinically indicated, breast MRI.

All patients who had undergone breast-conserving surgery (BCS) received bilateral mammography with craniocaudal (CC) and mediolateral oblique (MLO) views, whereas those who had undergone mastectomy underwent mammography of the contralateral breast only. Among 15,859 eligible cases, 454 women who developed CBC during surveillance were identified and designated as the CBC group. CBC was defined as any histologically confirmed carcinoma in situ or invasive breast carcinoma.

To establish a comparison group (control group), 454 women without any evidence of recurrence until their last follow-up examination were selected through 1:1 propensity score matching based on age and BI-RADS breast density. Nearest-neighbor matching without replacement was applied with a caliper of 0.2 standard deviations of the logit of the propensity score. Covariate balance after matching was verified using standardized mean differences, targeting SMD < 0.10 across covariates.

**Fig. 1:**
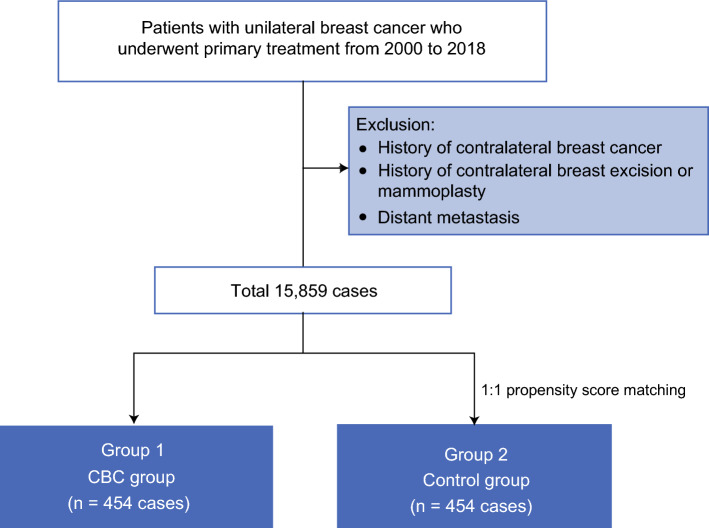
Study population flowchart. Patients with unilateral breast cancer who underwent primary treatment from 2000 to 2018 were included. Patients with a history of contralateral breast cancer, contralateral excision mammoplasty, or distant metastasis were excluded. The control group was matched 1:1 with the CBC group using propensity score matching, yielding 454 cases in each group

Exclusion criteria were as follows: prior bilateral breast cancer, prior contralateral excision, distant metastasis before CBC, and CBC diagnosed within 1 year of initial treatment. Patients meeting any of these criteria were excluded from both groups. After all exclusions and matching, 908 patients (454 per group) were included in the final analysis (Fig. 1).

### Mammogram Acquisition

All mammograms at our institution were performed using commercially available machines (Selenia Dimensions, Hologic, Bedford, MA; and Senographe 2000D FFDM, GE Medical System, Buc, France) and stored in the institutional picture archiving and communication system. Standard CC and MLO views were obtained according to institutional protocols.

For the CBC group, the most recent mammogram prior to the histologic diagnosis of CBC was selected for analysis. When CBC was visible on mammography, the image obtained at the time of recurrence was used; if the lesion was not mammographically visible and was instead detected by ultrasound or MRI, the most recent mammogram within the preceding 12 months was analyzed. For the control group, the latest available mammogram obtained at the last surveillance visit was used. In total, 454 digital mammograms from the CBC group and 454 from the control group were included as baseline images for AI-CAD analysis. All mammograms were anonymized before analysis to remove patient identifiers and ensure blinding.

### AI-CAD Assessment

All mammographic images were processed using a single commercially available AI-CAD system (Lunit INSIGHT MMG, version 1.1.7.3 and 1.1.8.1, Lunit Inc., Seoul, South Korea). The system has received regulatory clearance from the U.S., European and Korean Food and Drug Administration for clinical use in breast cancer detection.

This AI software processes digital mammograms at a pixel-level and automatically generates a heatmap highlighting suspicious regions with an abnormality score ranging from 0 to 100, where higher values indicate greater likelihood of malignancy. A prespecified decision threshold of 10 was used, corresponding to approximately 90% sensitivity in the system’s internal tuning dataset.^13^

For each patient, standard CC and MLO views of both breasts (four views in total) were analyzed when available; for patients who had undergone mastectomy, only two views of the contralateral breast were analyzed. The AI-CAD processed mammograms independently, without access to prior examinations or clinical information.

For each mammogram, the maximum abnormality score among all analyzed views was recorded as the per-patient AI abnormality score. An AI assessment was considered positive if the score exceeded 10 and the corresponding heatmap correctly localized the biopsy-proven site of CBC. Localization accuracy was verified by comparing the AI heatmap with the radiologist’s original clinical report and pathological findings.

### Reference Standards

The reference standard for CBC status was histopathologic confirmation obtained from biopsy or surgical specimens. For comparison with AI performance, the original mammographic assessments by breast radiologists at the time of imaging were used as the clinical benchmark.

Radiologists’ interpretations were based on Breast Imaging Reporting and Data System (BI-RADS) categories 1–5 documented in the clinical reports.^16^ BI-RADS categories 1 and 2 were classified as negative, whereas categories 4 and 5 were classified as positive. BI-RADS 3 assessments were considered positive for consistency with screening practice, as they require short-interval follow-up; however, cases that had undergone at least two consecutive 6-month follow-ups without interval change were reclassified as negative, reflecting routine clinical workflow. This approach was chosen to mirror real-world surveillance practice, in which BI-RADS 3 findings initially prompt heightened clinical attention but are subsequently regarded as benign when stability is confirmed over time.

All classifications were based on existing clinical reports without re-reading of mammograms to ensure that the comparator represented the actual diagnostic process in practice. Pathologic confirmation served as the final truth standard for all CBC cases, and both radiologist and AI evaluations were compared against this reference.

### Serial Mammogram Analysis for the CBC Group

For patients in the CBC group, all serial postsurgery surveillance mammograms obtained before the histologic diagnosis of CBC were additionally collected to investigate how early the AI-CAD system could identify abnormal findings suggestive of CBC on prior examinations. A total of 3,454 digital mammograms were analyzed, with a median of seven examinations per patient (interquartile range [IQR], 5–10).

All AI-analyzed images were independently reviewed by two board-certified breast surgical oncologists (J.-J.J. and E.K., each with 5 years of post-certification experience) to verify whether the AI-CAD system detected abnormal findings in earlier mammograms and, if detected, whether the heatmap correctly localized the eventual cancer site. Both reviewers were blinded to the clinical timeline.

To minimize recall bias, the order of cases was randomized prior to review, and all images were anonymized before evaluation. The lead time for each patient was defined as the interval (in months) between the AI-positive prior mammogram and the date of biopsy-proven CBC diagnosis.

### Statistical Analysis

The primary end points of the study were to investigate the sensitivity, specificity and the area under the receiver operating characteristics curve (AUC) of the AI-CAD software for detection of CBC. The sensitivity of AI assessments was compared with the assessment made by radiologists using the McNemar's test. Secondary analysis was performed to investigate how early the AI-CAD software could detect the abnormal signs of CBC on serial mammograms of each patient in the CBC group. All analyses were performed using R software version 3.6.3 (The R Foundation for Statistical Computing, Vienna, Austria).

## Results

### Patient Characteristics

A total of 908 patients (median age 53 years; IQR, 46–60) were included in the analysis. Most patients had dense breast tissue (BI-RADS breast density categories C and D): 546 (60.1%) were categorized as category C and 180 (19.8%) as category D. In our study, the control group was selected using 1:1 propensity score matching to ensure comparability with the CBC group. As a result, there were no significant differences in age and mammographic density between the two groups.

Among patients in the CBC group, 251 (55.3%) had invasive ductal carcinoma, 167 (36.8%) had ductal carcinoma in situ, and 23 (5.1%) had invasive lobular carcinoma. The remaining 2.9% (13 patients) included mucinous carcinoma and adenoid cystic carcinoma subtypes. Detailed clinicopathologic characteristics of CBC cases are provided in Table 1 and Supplemental Table S1.

**Table 1:** Patient demographics according to development of contralateral breast cancer

Number of women	All (*n* = 908)	CBC group (*n* = 454)	Control group (*n* = 454)	*p*
Median age*	53.0 [46.0–60.0]	53.0 [46.0–61.0]	53.0 [47.0–60.0]	0.995
Age category				1.000
Younger (≤50)	380 (41.9%)	190 (41.9%)	190 (41.9%)	
Older (>50)	528 (58.1%)	264 (58.1%)	264 (58.1%)	
BI-RADS breast density				1.000
A (fatty)	18 (2.0%)	9 (2.0%)	9 (2.0%)	
B (scattered density)	164 (18.1%)	82 (18.1%)	82 (18.1%)	
C (heterogeneously dense)	546 (60.1%)	273 (60.1%)	273 (60.1%)	
D (extremely dense)	180 (19.8%)	90 (19.8%)	90 (19.8%)	

*CBC* contralateral breast cancer

^*^Age at mammography

### Cancer Detection Performance of the AI-CAD Software

**Fig. 2:**
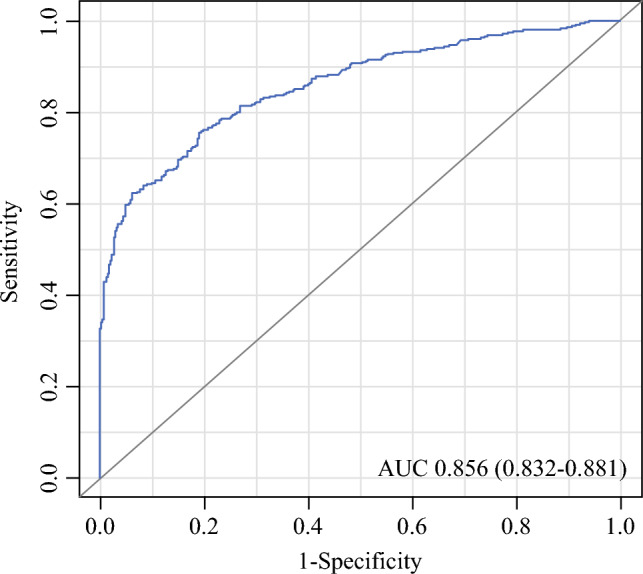
Receiver operating characteristics (ROC) curve of the AI-CAD system. ROC curve demonstrating the cancer detection performance of the AI-CAD system. The system achieved an area under the curve (AUC) of 0.856 (95% CI 0.832–0.881), highlighting its discriminative ability in identifying contralateral breast cancer

The AI-CAD system demonstrated robust diagnostic performance in detecting CBC on surveillance mammograms. The median AI abnormality score was significantly higher in the CBC group compared to the control group (23.5 [2.3–82.2] vs. 0.3 [0.1–1.5], *p* < 0.001) (Table 2). At the prespecified threshold of 10, the AI-CAD system correctly identified 271 of 454 CBC cases (sensitivity, 59.7%) and generated 28 false-positive findings among 454 controls (specificity, 93.8%). The corresponding AUC was 0.856 (95% CI 0.832–0.881) (Fig. 2). In a supplementary time-stratified analysis, AI-CAD demonstrated substantial detection rates over the study period (Table S2).

**Table 2:** Performance of the AI-CAD for detection of contralateral breast cancer

Number of examinations	CBC group (*n* = 454)	Control group (*n* = 454)	*p*
AI abnormality score	23.5 (2.3–82.2)	0.3 (0.1–1.5)	<0.001
AI diagnosis			<0.001
Positive	271 (59.7%)	28 (6.2%)	
Negative	183 (40.3%)	426 (93.8%)	

*CBC* contralateral breast cancer

Data are medians, with interquartile ranges in parentheses

Among the 28 false-positive assessments, 25 were interpreted as negative on radiologists’ baseline readings, one was recommended for digital breast tomosynthesis, and two were recommended for ultrasound, both of which yielded benign results on subsequent biopsy.

### Early Detection of Contralateral Breast Cancer by the AI-CAD Software

**Fig. 3:**
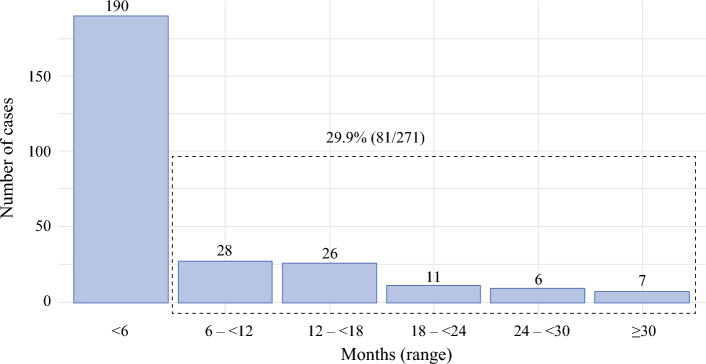
Early detection of contralateral breast cancer by AI-CAD system over time. Timeline showing the early detection of contralateral breast cancer (CBC) by the AI-CAD system. The AI-CAD identified 29.9% (81/271) of CBC cases on earlier mammograms, with a median detection time of 13.3 months before biopsy-confirmed diagnosis. Notably, 10.3% of cases were detected more than 6 months earlier, and 19.6% were identified more than a year before clinical diagnosis. This highlights the AI-CAD system's capability to detect early signs of CBC, potentially improving early intervention strategies

Among the 271 CBC cases detected by the AI-CAD, 81 (29.9%) showed abnormal findings on prior surveillance mammograms before the biopsy-proven diagnosis (Fig. 3). The median lead time was 13.3 months (IQR, 9.6–19.9 months) between the earliest AI-positive mammogram and the date of pathologic confirmation.

Of these 81 early-detected cases, 28 (10.3%) were identified more than 6 months before clinical diagnosis, and 53 (19.6%) were detected more than one year earlier. All AI-positive findings on prior mammograms were anatomically consistent with the eventual site of CBC on subsequent imaging and histopathology.

An illustrative case of early AI detection is shown in Supplemental Fig. S1, in which the AI-CAD system correctly localized an abnormality 2 years before it was recognized radiographically and confirmed by biopsy.

### Enhanced Detection of Contralateral Breast Cancer with the AI-CAD Software

Given that conducting both mammography and ultrasound simultaneously during surveillance is common in South Korea and recognizing that a significant proportion of CBC cases were missed by mammography but detected by other imaging modalities, we analyzed the clinical diagnosis methods of the 454 CBC cases.

In the clinical setting, mammography detected 243 of 454 CBC cases (53.5%), either alone or in combination with adjunctive ultrasound. The remaining 211 of 454 cases (46.5%) were missed on mammography but identified through other modalities, such as ultrasound, MRI, CT, magnification views, or clinical examination (Table S3).

When directly compared with radiologists’ baseline mammographic assessments, the standalone AI-CAD system achieved a higher sensitivity (59.7% vs. 53.5%), representing an absolute improvement of 6.2%. Using McNemar’s test with continuity correction, this difference was statistically significant (*χ*^2^ = 6.88; *p* = .009), although the clinical impact of this difference cannot be assessed.

**Fig. 4:**
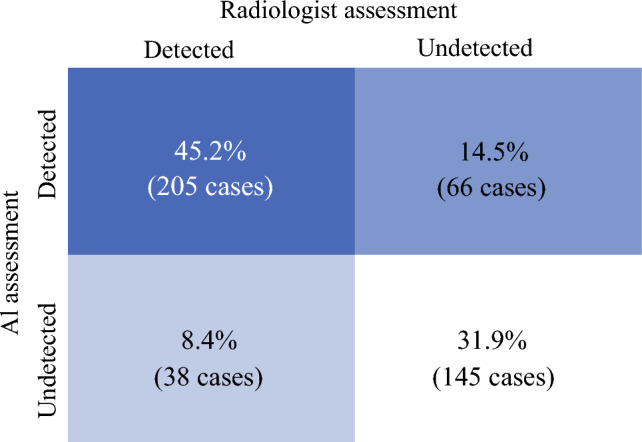
Comparison of detection sensitivity: AI system vs. radiologist in identifying abnormalities from mammograms. Comparison of detection outcomes between the AI-CAD system and radiologists on surveillance mammograms for contralateral breast cancer (CBC). AI-CAD showed higher sensitivity than baseline radiologist assessments (59.7% vs. 53.5%, *p* = 0.009). Concordant interpretations were observed in 350 of 454 cases (77.1%), including 205 positives and 145 negatives, while discordant results occurred in 104 cases (22.9%), with 66 (14.5%) detected only by AI-CAD and 38 (8.4%) detected only by radiologists, suggesting potentially complementary detection patterns

The AI-CAD system and radiologists showed concordant results in 350 of 454 cases (77.1%), including 205 concordant positives and 145 concordant negatives. Discordant interpretations were observed in 104 of 454 cases (22.9%), among which 66 (14.5%) were detected only by AI and 38 (8.4%) were detected only by radiologists (Fig. 4). This highlights the AI software’s capability to identify potential abnormalities that might be less obvious to human readers.

The distribution of AI abnormality scores differed significantly across detection scenarios (Fig. S2). Median scores were higher for lesions detected by both AI and radiologists (80.9 [IQR, 47.4–96.8]) than for those detected by AI alone (33.4 [IQR, 18.1–73.7]; *p* < .001).

### Clinicopathologic Characteristics of CBC by AI Versus Radiologist Detection

Clinicopathologic features of the 309 CBC cases detected on mammography were compared across three detection scenarios: AI only (*n* = 66), radiologist only (*n* = 38), and both AI and radiologists (*n* = 205) (Table 3). Cases detected by radiologists but missed by AI were more frequently observed among women with a history of breast-conserving surgery (BCS) (*p* = 0.025).

**Table 3:** Characteristics of detected contralateral breast cancer on mammogram by different detection scenarios

	AI(+), radiologist (+) (*n* = 205)	AI(+), radiologist (−) (*n* = 66)	AI(−), radiologist (+) (*n* = 38)	*p*
Type of breast surgery				0.025
Breast-conserving surgery	108 (52.7%)	27 (40.9%)	26 (68.4%)	
Mastectomy	97 (47.3%)	39 (59.1%)	12 (31.6%)	
History of chemotherapy				0.663
Yes	132 (64.4%)	40 (60.6%)	23 (60.5%)	
No	65 (31.7%)	23 (34.8%)	15 (39.5%)	
Unknown	8 (3.9%)	3 (4.5%)	0 (0.0%)	
History of endocrine therapy				0.085
Yes	125 (61.0%)	30 (45.5%)	22 (57.9%)	
No	80 (39.0%)	36 (54.5%)	16 (42.1%)	
BI-RADS breast density				0.688
A	7 (3.4%)	1 (1.5%)	0 (0.0%)	
B	41 (20.0%)	15 (22.7%)	5 (13.2%)	
C	121 (59.0%)	40 (60.6%)	27 (71.1%)	
D	36 (17.6%)	10 (15.2%)	6 (15.8%)	
Median age at CBC	54.0 [47.0–62.0]	54.5 [47.0–64.0]	52.0 [43.0–59.0]	0.157
Histologic type of CBC				0.056
Ductal carcinoma in situ	73 (35.6%)	21 (31.8%)	10 (26.3%)	
Invasive ductal carcinoma	114 (55.6%)	36 (54.5%)	27 (71.1%)	
Invasive lobular carcinoma	8 (3.9%)	8 (12.1%)	1 (2.6%)	
Other invasive subtypes	10 (4.9%)	1 (1.5%)	0 (0.0%)	

*CBC* contralateral breast cancer

Although not statistically significant, the histologic subtype distribution differed among detection groups (*p* = 0.056). Invasive lobular carcinoma was proportionally more common in AI-only detected cases, whereas invasive ductal carcinoma was predominant in radiologist-only detections.

No significant differences were observed across detection groups in terms of age at CBC diagnosis (*p* = 0.157), prior chemotherapy (*p* = 0.663), endocrine therapy (*p* = 0.085), or mammographic breast density (*p* = 0.688).

These findings suggest that AI-CAD may be particularly sensitive to subtle morphologic patterns such as ILC, whereas radiologists may better recognize structural distortions associated with post-surgical changes after BCS.

## Discussion

In this study, we demonstrated that AI-CAD software can be an effective diagnostic tool to screen CBC in women with a history of breast cancer. The AI-CAD software showed robust diagnostic performance, achieving a sensitivity of 59.7% and a specificity of 93.8%, with an AUC of 0.856. Importantly, AI-CAD demonstrated its potential to improve CBC detection, identifying 14.5% of CBC cases that were missed by radiologists and detecting 29.9% of cases early, with a median lead time of 13.3 months before biopsy indication. Overall, these findings suggest that incorporating AI-CAD software into clinical practice could significantly improve mammographic diagnosis in breast cancer survivors.

The main difference between radiologists and AI-CAD software for mammographic interpretation is that radiologists routinely compare bilateral four-view (CC and MLO) images with previous images to identify changes over time, whereas most AI-CAD software do not incorporate historical images. Published studies have highlighted this as a limitation of AI, suggesting the need for longitudinal analysis.^9,11,17^ However, our study utilized an AI-CAD software that analyzes each image independently and still demonstrated higher sensitivity. This suggests that AI algorithms, trained on large datasets, can effectively identify abnormal features in single-instance mammograms. Supporting this, studies by Wu et al.^18^ and Kim et al.^13^ highlighted the robust image recognition capabilities of AI-CAD software and their effectiveness in initial screenings without prior images. Our study further demonstrated that even when annual follow-ups provided historical mammograms for radiologists, the AI-CAD software could effectively detect abnormalities without needing previous or bilateral images.

When previous mammograms were available for CBC cases, the AI-CAD software was able to detect approximately 30% of cases in earlier mammograms. Specifically, 81 of 271 CBC cases detected by the AI-CAD software were recognized in prior mammograms. Notably, the AI-CAD identified 28 cases (10.3%) more than 6 months earlier, and 53 cases (19.6%) more than a year earlier. This early detection capability suggests potential benefit of AI-CAD in reducing missed cancer and interval cancer rates among patients with history of breast cancer, consistent with previous studies that AI can be used as a decision support or triage for radiologists,^8,18^ and AI could detect interval cancers classified as negative at screening.^19^ The early detection is critical as it can potentially improve patient outcomes and survival rates. However, it is important to note that identifying abnormalities in the absence of clinical signs may cause unnecessary anxiety for the patient. Therefore, further discussions and studies are needed to evaluate the clinical and economic implications of early detection.

A comparison of CBC cases across different detection scenarios showed that those missed by AI but detected by radiologists on mammograms had a significantly higher proportion of women with a history of BCS. This finding may indicate potential limitations in using the current AI-CAD software, which was developed for the general population, for screening women with a history of BCS. It is important to note that the Lunit INSIGHT MMG was developed to analyze four bilateral views together in screening mammograms, and its performance was superior with four-view input compared to single-view input. In our study, the four views from women with bilateral mammography included images with surgical scars, which may have adversely affected the algorithm's performance, potentially leading to a higher number of missed cases in BCS patients. Conversely, for patients who had undergone mastectomy, the two-view images were analyzed independently, making it challenging to achieve the high performance generally expected from four-view analyses. Therefore, it is crucial to emphasize as a limitation that the algorithm was not trained specifically for the purpose of this study, and further research is necessary to understand the underlying reasons for missed CBC cases and to refine AI algorithms to better fit for surveillance mammography.

Although the difference did not reach statistical significance, it is notable that cases detected by AI but missed by radiologists had a markedly higher proportion of ILC. ILC is often more subtle on imaging, and its extent is frequently underestimated in radiologic assessments.^20^ This raises the possibility that AI-CAD may have an advantage in identifying such subtle patterns that are challenging for conventional assessment. Future studies are needed to validate whether AI is indeed more sensitive to ILC and to explore the mechanisms behind this potential strength.

Additionally, an important limitation of this study is its single-center retrospective design, with nearly all participants being of Asian ethnicity. This demographic homogeneity limits the generalizability of the findings to more diverse populations. Patients were generally younger and had denser breast tissue, which are known factors that can complicate mammographic detection. Our missed case rate was notably high at 31.9%, compared with an earlier multinational study by Kim et al.,^13^ which reported an 8% missed rate for both AI and radiologists in screening mammograms, and a study by Yoen et al.,^21^ which reported a 22.1% missed rate in screening-detected breast cancers. While our study provides valuable insights about AI-CAD within this specific demographic, prospective studies including multicenter, multiethnic evaluations are essential to ensure the AI software's robustness and reliability in diverse clinical settings, enhancing its global applicability in breast cancer surveillance. Moreover, our study did not directly compare the effectiveness of standalone AI with radiologists in detecting CBC, as all prior radiologic assessments were conducted alongside sonographic evaluations. Future research should aim to evaluate mammography independently to assess strengths and limitations of AI-CAD software when utilized within guideline-based surveillance recommendations.

In summary, our findings suggest that AI-CAD, even when developed for screening populations, may provide added value in surveillance mammography for breast cancer survivors by facilitating earlier detection of contralateral breast cancer. While the clinical and economic implications of earlier detection require further evaluation, these results support the potential role of AI-CAD as a complementary decision-support tool. Prospective, multicenter studies in diverse populations are warranted to validate its clinical utility.

## Supplementary Information

Below is the link to the electronic supplementary material.

Supplementary file1 (DOCX 895 KB)
