# Supplementary material for: Enhancing Early Detection of Contralateral Breast Cancer in Breast Cancer Survivors Using AI-Assisted Mammography
Source: Ann Surg Oncol. 2026 Mar 6;33(6):5581–9. doi: 10.1245/s10434-026-19348-z (PMC13179211; doi:10.1245/s10434-026-19348-z)
Supplement: Supplementary file 1 — Supplementary file1 (DOCX 895 KB) [file 10434_2026_19348_MOESM1_ESM.docx]

**SUPPLEMENTAL MATERIALS**

**Table S1. Histologic subtypes of contralateral breast cancer**

| **Diagnosis** | **Number of Patients (%)** |
| --- | --- |
| Ductal carcinoma in situ | 167 (36.8%) |
| Invasive ductal carcinoma | 251 (55.3%) |
| Invasive lobular carcinoma | 23 (5.1%) |
| Other invasive subtypes* | 13 (2.9%) |

*Includes mucinous carcinoma and adenoid cystic carcinoma

**Table S2. Performance of AI-CAD and radiologist mammographic detection across different time periods**

|  | **2004–2010 (n=54)** | **2011–2017 (n=176)** | **2018–2023 (n=224)** | **P value** |
| --- | --- | --- | --- | --- |
| **AI abnormality score** | 45.1 (3.3–89.2) | 18.2 (1.1–75.4) | 24.1 (3.1–84.6) | <0.001 |
| **AI diagnosis** |  |  |  | <0.001 |
| Positive | 38 (70.4%) | 97 (55.1%) | 136 (60.7%) |  |
| Negative | 16 (29.6%) | 79 (44.9%) | 88 (39.3%) |  |
| **Radiologist assessment** |  |  |  | <0.001 |
| Detected | 41 (75.9%) | 86 (48.9%) | 116 (51.8%) |  |
| Not detected | 13 (24.1%) | 90 (51.1%) | 108 (48.2%) |  |

**Table S3. Diagnostic modalities for contralateral breast cancer: AI-CAD versus clinical diagnosis**

|  | **Confirmed CBC (n=454)** | |
| --- | --- | --- |
|  | **Number** | **Sensitivity** |
| **AI-CAD** |  |  |
| Detected by MG | 271 | 59.7% |
| **Clinical diagnosis** |  |  |
| Detected by MG | 243 | 53.5% |
| MG+ | 68 | - |
| MG and US+ | 175 | - |
| Detected by other modality (undetected by MG) | 211 | 46.5% |
| US+ | 191 | - |
| MRI+ | 15 | - |
| CT+ | 2 | - |
| CBE+ | 2 | - |
| Magnification MG+ | 1 | - |

CBC: contralateral breast cancer, MG: mammography, US: ultrasound, MRI: magnetic resonance imaging, CT: computed tomography, CBE: clinical breast examination


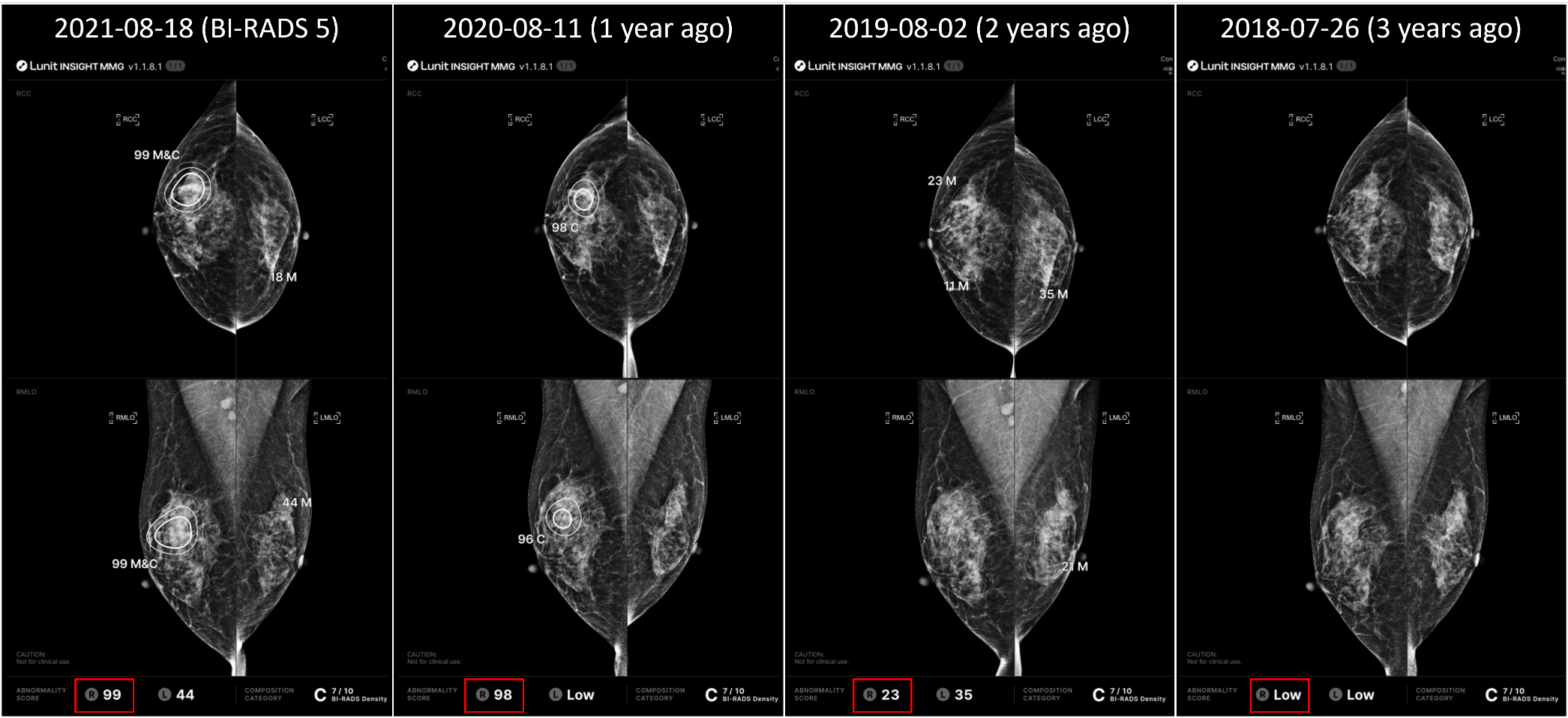


**Figure S1. Early detection of contralateral breast cancer by AI-CAD system**

This figure illustrates a case in which the AI-CAD system identified contralateral breast cancer in the right upper outer quadrant two years prior to its pathological confirmation in 2021. The system showed progressively increasing abnormality scores over time, highlighting its potential to detect cancer earlier than conventional mammographic interpretation.


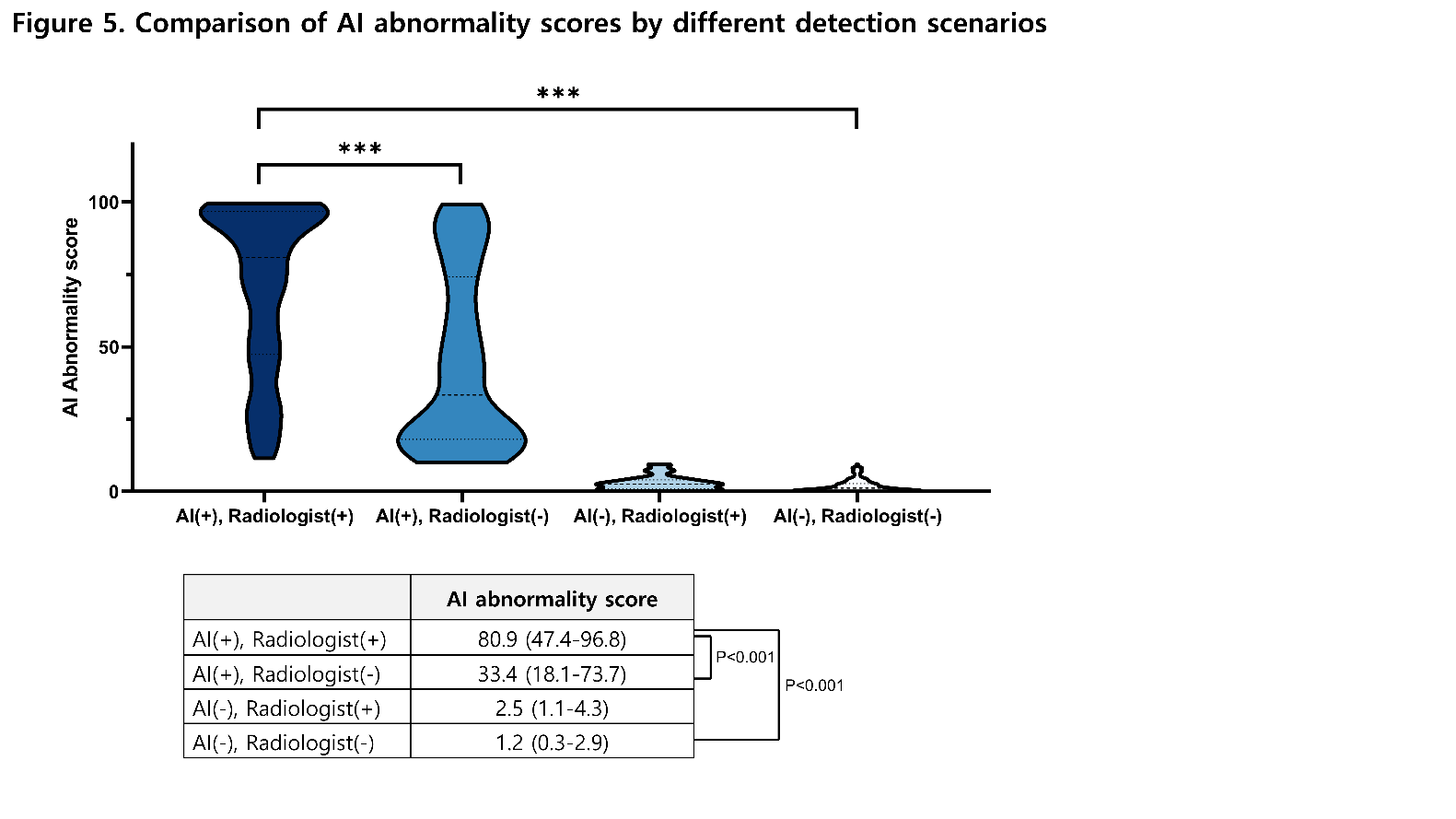


**Figure S2: Comparison of AI Abnormality Scores by Different Detection Scenarios**

Distribution of AI abnormality scores based on different detection scenarios. Cases detected by both AI and radiologists had significantly higher AI abnormality scores compared to cases detected only by AI. Cases detected only by radiologists or missed by both had much lower scores, highlighting differences in AI scores across various detection outcomes (p<0.001).
